# Using Exponential Random Graph Models to Analyze the Character of Peer Relationship Networks and Their Effects on the Subjective Well-being of Adolescents

**DOI:** 10.3389/fpsyg.2017.00583

**Published:** 2017-04-13

**Authors:** Can Jiao, Ting Wang, Jianxin Liu, Huanjie Wu, Fang Cui, Xiaozhe Peng

**Affiliations:** ^1^College of Psychology and Sociology, Shenzhen UniversityShenzhen, China; ^2^The Faculty of Humanities and Social Sciences, City University of MacauMacau, Macau

**Keywords:** peer relationships, subjective well-being, exponential random graph models, social network analysis

## Abstract

The influences of peer relationships on adolescent subjective well-being were investigated within the framework of social network analysis, using exponential random graph models as a methodological tool. The participants in the study were 1,279 students (678 boys and 601 girls) from nine junior middle schools in Shenzhen, China. The initial stage of the research used a peer nomination questionnaire and a subjective well-being scale (used in previous studies) to collect data on the peer relationship networks and the subjective well-being of the students. Exponential random graph models were then used to explore the relationships between students with the aim of clarifying the character of the peer relationship networks and the influence of peer relationships on subjective well being. The results showed that all the adolescent peer relationship networks in our investigation had positive reciprocal effects, positive transitivity effects and negative expansiveness effects. However, none of the relationship networks had obvious receiver effects or leaders. The adolescents in partial peer relationship networks presented similar levels of subjective well-being on three dimensions (satisfaction with life, positive affects and negative affects) though not all network friends presented these similarities. The study shows that peer networks can affect an individual’s subjective well-being. However, whether similarities among adolescents are the result of social influences or social choices needs further exploration, including longitudinal studies that investigate the potential processes of subjective well-being similarities among adolescents.

## Introduction

In adolescence, and with increasing physical and cognitive development, a child’s psychological awareness begins to resemble that of an adult. Adolescents spend more and more time with their contemporaries, especially their peers. A peer relationship is the relationship of a common activity and mutual cooperation among children in the same or similar age group, but mainly refers to a relationship between peers or individuals at a similar level of psychological development, which is built and developed through communication (Yang, 2008). And peer relationships are the main sources of social support for adolescents and the main driving force in enhancing an adolescent’s self-concept and well-being (Furman and Buhrmester, 1992). Good peer relationships cannot only promote the development of an adolescent’s social cognition and social skills, but also improve their physical and mental health and enhance their subjective well-being. Negative peer relationships not only hinder an adolescent’s academic performance and personality development, but might lead to emotional problems such as anxiety, depression, and mental illness (Chen and Zhou, 2007). Adolescent school-based networks are important for developing these peer relationships (Haas et al., 2010). Researchers have argued that peer relationships may promote the development of an adolescent’s self-identity through social comparison and symbolic evaluation (Brown and Lohr, 1987). Adolescents see their peer groups as typical models for their views and behaviors, and use the identity of the peer groups to regulate their own behavior. In this way, adolescents develop similarities to others in their groups. According to social communication theory, in a social network, a person’s emotions, opinions, and behaviors are like an epidemic and can spread by social interaction (Kiuru et al., 2012). And according to similarity theory, individual similarities in values, characteristics and behaviors increase predictably, which enables them to share the same feelings and develop a sense of belonging, and makes them easy to get along with (Batool and Malik, 2010).

The developmental literature has long emphasized the strong role of peer groups in determining our inclination toward social behaviors (Brown, 2004). Many researchers have carried out a social network analysis of adolescents especially in relation to their social behaviors. Researchers have studied the relationship between peer-related physical activity social networks (Voorhees et al., 2005) and peer aggression (Low et al., 2013); health (Haas et al., 2010); obesity (Marqués et al., 2015); smoking (Ennett and Bauman, 1993; Lakon et al., 2013; Lambert, 2014); substance use (Ennett et al., 2006); and drinking (Mundt, 2011; Deutsch et al., 2014). However, whether they like their lives is rarely explored through this method. Our investigation aims to satisfy curiosity about a child’s inner state.

Subjective well-being is a personal evaluation of an individual’s overall living conditions. In other words, subjective well-being is how much a person likes his or her life (Veenhoven, 2013) and in colloquial terms is sometimes labeled “happiness.” This is a multidisciplinary research field. The social psychologist Diener, one of the few internationally recognized academic authorities in this area, pointed out that the subjective well-being of the individual produces a positive attitude and positive feelings by comparing the actual state of life with ideal life. Subjective well-being is characterized by subjectivity, initiative and comprehensiveness (Diener, 2000). The evaluation criteria are made by an individual’s own standards without reference to any external evaluation criteria. The evaluation criteria have the characteristics of subjectivity, stability and integrity. External factors such as gender, age, income and life events, as well as internal factors such as personality, self-esteem, self-efficacy, and self-concept, all have an impact on subjective well-being (Diener et al., 1995, 1999; McCullough et al., 2000; Schimmack and Diener, 2003; Ferrer-i-Carbonell, 2005; Gutiérrez et al., 2005; Gilman and Huebner, 2006; Karademas, 2006; Zhou et al., 2012; Ulloa et al., 2013). Since subjective well-being is the main aspect of living quality and has a close relationship with mental health, studies of subjective well-being have been highly valued (Diener, 1984). Meanwhile, various studies have shown that peer/interpersonal relationships and subjective well-being are related to a certain degree (Hussong, 2000; Liu and Gong, 2000; Dai, 2005; Demir and Weitekamp, 2007; Litwin and Shiovitz-Ezra, 2010). In addition, the former has a strong predictive ability on the latter (Dai, 2005; Chen, 2006; Demir et al., 2007; Xia, 2007; Wu, 2008; Zhang and Zhu, 2012).

The period known as adolescence is a critical period for psychological development. Psychological symptoms such as low subjective well-being have been recognized as being common (La Greca and Lopez, 1998). These obstacles can last a long time, often beginning in adolescence and extending to adulthood (Devine et al., 1994), and are likely to become risk factors for adult mental disorders (Devine et al., 1994; Aalto-Setälä et al., 2002). Therefore, studies of the relationship between adolescent peer relationships and subjective well-being have a certain practical significance.

Although there has been much fruitful research on the relationship between peer relationships and subjective well-being, as outlined above, there is a common problem with these studies; that is, their methodological premise in applying statistical analysis. Specifically, researchers have presented relational data in a simplified form as attribute data. Peer relationships in essence are relational attributes and reflect interpersonal relationships as well as interdependencies between individuals. However, if peer relationships are regarded as attribute data, then the methods used to conduct statistical analysis on the basis of this assumption would be those that are relevant to attribute data only, such as correlation analysis and regression analysis. The statistic type is clearly against the premise of these methods in this context, and the conclusions drawn from such research may not be valid (Jiao et al., 2014).

Social network analysis is an effective method of solving this problem. As a method for dealing with relational data, social network analysis fully considers the impacts of the social situation on individual behaviors and focuses on the relationship between individuals. The social context is constituted by the relationship between individuals. Relationships constitute a network. In social networks, the points (or nodes) represent the units such as individuals, families, organizations, and social groups. The edges represent whether a relationship between points exists and its strength. By network analysis, the relationships between individuals can be described and measured. Additionally, the resources and information within the relationship can also be described and measured. Furthermore, a model can be built for these relationships, which can be used to study the interactions between these relationships and individual behaviors (Liu, 2004).

Exponential Random Graph Models (ERGMs) are a method of social network analysis for building complex social network structures (Robins et al., 2007). The model assumes that the emergence of a relationship might be influenced by the presence or absence of other relationships and/or individual attributes (Robins et al., 2007). Compared with other social network analysis models, an ERGM focuses on the interaction between the structures of a relationship network (such as reciprocity, transitivity, and popularity) and individual attributes (such as gender and education level). In order to understand the inner mechanism of peer relationships and subjective well-being more clearly, this study used an ERGM model to explore the relationships between network structures in adolescent peer relationships and the individual’s subjective well-being.

Our purpose is to clarify the character of a peer relationship network and the mechanism of the peer relationship influence on subjective well-being. Studies have shown that there are effects of reciprocal structure, transitivity structure, popularity structure and expansiveness structure in peer networks (Schaefer et al., 2010; Daniel et al., 2013). Thus, this study assumes the following.

• Hypothesis 1: There are significant reciprocal structure effects in adolescent peer networks.
• Hypothesis 2: There are significant transitivity structure effects in adolescent peer networks.
• Hypothesis 3: There are significant popularity structure effects in adolescent peer networks.
• Hypothesis 4: There are significant expansiveness structure effects in adolescent peer networks.

Adolescents and their friends might have similarities in a variety of social, behavioral, and psychological characteristics (Prinstein and Dodge, 2008). Numerous studies have found that adolescent friends have similarities in externalizing problems such as attacks (Sijtsema et al., 2010); internalizing problems such as depression (Van Zalk et al., 2010); health risk behaviors such as smoking (Mercken et al., 2010); “happy” emotions (Fowler and Christakis, 2008); and prosocial behaviors (Barry and Wentzel, 2006). Thus, this study also assumes the following.

• Hypothesis 5: Adolescents show similar levels among peers in each dimension of subjective well-being (satisfaction with life, positive affects and negative affects).

Receiver effects build relationships between individual attribute variables and popularity. Popularity refers to the nominated numbers that individuals receive from other individuals in their network class. More nominated numbers indicate that the individual is more popular. Studies have shown that popular individuals prefer interactions with their peers and have more positive attitudes. They are seldom isolated, refused or repelled by peers. They experience more positive affects and have fewer negative experiences (LaFontana and Cillessen, 2002; Wen, 2011). Thus, this study further assumes:

• Hypothesis 6: There is a significant positive receiver effect in the positive affective dimension of subjective well-being; namely, individuals who have higher levels of positive affect will have more friends and be more popular, and vice versa.
• Hypothesis 7: There is a significant negative receiver effect in the negative affective dimension of subjective well-being, namely, individuals who have higher levels of negative affect will have fewer friends and be less popular, and vice versa.

## Materials and Methods

### Participants

Nine junior middle schools were selected at random from Shenzhen, China. Twenty-nine classes were then selected at random from these nine schools (mean classes 3.22, standard deviation 0.44). There were 1,497 students altogether. All students were given questionnaires which were handled as follows. First, the questionnaires that did not meet requirements were excluded from the study. These included cases of: (a) multiple answers (a participant provided more than one option for an item); (b) no answers (a participant failed to provide an option for an item); and (c) regular answers (a participant provided the same option or a regular array of options for a series of items). Secondly, the subjects that obtained subjective well-being (SWB) scores exceeding three standard deviations were removed from the study, by which the influence of extreme values was eliminated. Thirdly, the subjects who were at the edge or on the periphery of a class network were excluded from the study as these subjects did not associate with other members in their class network and became isolated points. Finally, there were 1,279 subjects remaining, including 678 boys and 601 girls. The study was conducted in accordance with the Declaration of Helsinki and was approved by the Academic Committee of the College of Psychology and Sociology, Shenzhen University. All participants (or the parents of participants who were under the age of 16) provided written informed consent of participation in the study.

### Research Tools

#### The Questionnaire: Peer Nomination

The questions in the questionnaire were designed as follows. As regards peer nomination, participants were asked: “Please write the name of your best friends in the class (at least three)” (Lubbers and Snijders, 2007). In measuring peer relationships, if a member nominated another member it meant there was a relationship between them, which was then recorded as 1 in the relationship matrix; otherwise it was recorded as zero.

#### Subjective Well-being Scale

The SWB scale was built from two constituents: the Satisfaction with Life Scale (SWLS) and the Affect Balance Scale (ABS). The SWLS scale was compiled by Diener et al. (1985). It has been used to measure the cognitive dimension of subjective well-being (Zhu et al., 2012). The scale contains five items, each of which employs 7 score grades from “strongly disagree” to “strongly agree.” Strongly disagree is recorded with a score of 1, while strongly agree is recorded with a score of 7, the scores increasing in sequence. Higher scores indicate a higher satisfaction with life (SWL), whilst a low score means a low SWL. The Alpha coefficient of the scale is 0.78. The split-half reliability is 0.70. The fit index of confirmatory factor analysis is, respectively, the ratio of (chi-square/degrees of freedom) that is 6.71, RMSEA = 0.071, GFI = 0.97, and CFI = 0.96, which shows that the structure of the scale has a good validity (Xiong and Xu, 2009). The internal homogeneity coefficient α of the measurement is 0.77 and meets metrological standards.

The ABS scale was compiled by Bradburn and Noll (1969). The reliability estimates of Bradburn’s original study showed acceptable reliability coefficients (Bradburn and Noll, 1969). ABS has been used to measure the affective dimension of subjective well-being such as positive affect and negative affect (Yue et al., 2006). Our study uses the Chinese version of the Mental Health Assessment Scale, which was collected by Wang et al. (1999). The scale contains 10 items, which are used to describe a person’s feelings in the past few weeks. Among them, five items describe positive affects and five items describe negative effects. The answer “Yes” is recorded with a score of 1; the answer “No” with a score of 0. A higher score indicates a higher frequency of experiencing positive or negative effects (Zhang et al., 2007). In the scale, the retest reliability of positive affects and negative effects are both above 0.80; additionally, the correlation values between these two subscales is less than 0.10, which indicates the validity of the scale and its reliability (Ou et al., 2009). In the measurement, the internal homogeneity coefficient α of the positive affective dimension is 0.60, and the internal homogeneity coefficient α of the negative affective dimension is 0.63. The ABS has been translated into many languages including Cantonese, Vietnamese, and Laotian, and a cultural equivalence has been found (Devins et al., 1997). The original version of ABS and the Chinese version have both been shown to be reliable, and the measurement meets metrological standards.

### Exponential Random Graph Models

Exponential Random Graph Models (ERGMs), also known as *P*^∗^ models, were proposed by Frank and Strauss (1986) to explain a series of statistical models in social networks. An ERGM can infer how network relationships are formed. The model does not focus on predicting individual outcome variables in the network but focuses on the formation of deductive relations. It takes the network as a graph constituted by nodes (actors) and edges (relationships). It inspects the probability distribution of the set of all graphs with a fixed number of points (or nodes) (Ma et al., 2011). The model assumes that the network was generated at random. The probability of the observed graph depends on the number of occurrences of various structures in the model (Wang et al., 2009). Its basic form is as follows:

$$\text{Pr}(X=x)=\frac{\text{exp}\left\{\theta^{\prime }z\left(x\right)\right\}}{k\left(\theta \right)}=\frac{\text{exp}\left\{\theta_{1}z_{1}\left(x\right)+...+\theta_{r}z_{r}(x)\right\}}{k\left(\theta \right)}$$

In the above formula, the meaning of each expression is as follows (Ma et al., 2011). Pr(*X* = *x*) is the probability of some actual relationship between individuals. *θ* is a series of network structure parameter vectors, including reciprocal structure parameters, transitivity structure parameters and star-shaped structure parameters. *z(x)* is a series of network statistic vectors. The series contains not only particular network structure parameters (such as reciprocal parameters, transitivity parameters and star-shaped structure parameters), but also attribute parameters of the actors in the network (such as grade, gender, and attitudes). *k* is a constant and guarantees that the probability distribution is a normal distribution.

The dependence assumption is the basic theoretical assumption of ERGMs. “My friend’s friend is my friend” is a typical dependence assumption in a social network. The assumption is that the existence of some relationships will produce, maintain or destroy other relationships (Robins, 2011). If a relationship does not rely on other relationships, it can be said that the existence of some relationships will affect the existence of other relationships to some extent, and that they have no incentive to form the structure. The dependence assumption is presented by specific structures which reflect how relationships are generated in the network. The network structure is a small network mode, which is constituted by points (or nodes) and the relationships among points (Brughmans et al., 2014). **Table 1** presents the common structural parameters of ERGMs for a directed network.

**Table 1:** The common structural parameters of exponential random graph models for a directed network.

Parameter	Structure of point	Description of structure
Reciprocity	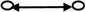	Two points select each other as friends, which creates a reciprocal relationship.

Transitivity	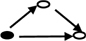	My (black point) friend’s (white point) friend (white point) is also my friend.

Popularity	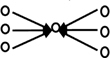	The points on both sides all select the central point as a friend. The central point is the most popular one.

Expansiveness	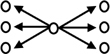	The central point selects the points on both sides as friends. The central point is actively making friends with other points.

Receiver	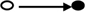	The relationship is sent from any point (white point) to some specific attribute point (black point).

Sender	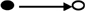	The relationship is sent from some specific attribute point (black point) to any point (white point).


*The point represents an “actor”; the directed line represents a “directed relationship from sender to receiver.”*

The reciprocity assumption is that if actor A selects actor B, then actor B will also select actor A. Reciprocity is a basic characteristic of social life and has been proved in peer groups (Snyder et al., 1996; Strayer and Santos, 1996). Popularity means that an individual has a higher “in-degree” compared with other individuals in the network (“in-degree” counts the total number of actors who select a particular individual). Higher in-degrees show that some people are more attractive than others, and the popularity assumption is that an individual will select the one actor that others have all selected to make friends (Barabási and Albert, 1999; Gould, 2002). Transitivity measures triangular closure trends in the network; namely, “my friend’s friend is also my friend.” If transitivity appears in peer groups, the reason might be that more and more individuals are willing to share each other’s friends, or might be due to a psychological need for balance (Daniel et al., 2013).

Based on various dependence assumptions, ERGMs contain several kinds of models. The simplest one is the Bernoulli Graph Model, which assumes that the edges and binary relations are independent in all networks. Therefore, there are only edges and no other structures in a Bernoulli Graph Model. A Dyadic Model assumes that the dyadic relations in a directed network graph are all independent. If the relationship between actor A and actor B is irrelevant to the relationship between actor B and actor C, there are two structures in the model: edges and reciprocated edges (Wang et al., 2009). A reciprocated edge means that if actor A selects actor B, then actor B will also select actor A.

However, the two models above are unrealistic, both from a theoretical standpoint and from practical experience. Therefore, Markov independence was introduced by Frank and Strauss (1986); this assumes that the relationship between actor A and actor B depends on any other relationships related to A or B. Under this condition, if there is a common actor in two relationships, then the relationships should be considered as conditionally independent (Robins et al., 2007). The Markov Random Graph Model was proposed based on this Markov dependence assumption. A Markov Random Graph Model contains not only the structural parameters of edges and reciprocated edges, but also various “two-star” structural parameters (where two actors both have relationships with a third actor). The structural parameters of “two-out-star” (an actor simultaneously selects two other actors as friends) are related by expansiveness. The structural parameters of “two-in-star” (an actor is selected by two other actors simultaneously as a friend) involves popularity. The simplest Markov Random Graph Model is a two-star model, which has only edges and two-stars within its structure. Researchers subsequently noted the importance of transitivity and cyclicity, and brought further structural parameters into the Markov Random Graph Model (Newman, 2003). The expanded model contains higher star-shaped statistics such as three-star structures (where three actors all have relationships with a fourth actor).

However, this model can only fit the data in quite limited circumstances. Additionally, many studies have shown that the model will have gradual degradation problems when it is estimated and simulated. A Markov Random Graph Model was therefore not considered to be a good model for observing social networks (Pattison et al., 2007). Researchers then introduced the concept of partial conditional dependence and proposed a Realization-Dependent Model (e.g., Snijders et al., 2006). This model assumes that if there is a relationship between two actors, then it can be regarded as partially conditionally dependent. The model has three new statistics: namely, alternating k-star (k actors all have a relationship at the same time with actor k + 1); alternating k-triangles (two actors with a relationship build triangular relationships with k actors); and alternating independent two-paths (two independent actors build two-path relationships with multiple third party actors). The convergence of the model is effectively improved with these additions.

In summary, the Bernoulli assumption is unsuitable for real network data. Although the Markov independent assumption broadens the network structure, the model might degrade for smaller networks. Partial conditional dependence assumptions enable us to build network aggregation effects and are closer to real social networks. Consequently, the realization-dependent model has been widely applied by scholars.

The common parametric estimation methods of ERGMs are the Maximum Pseudo-Likelihood Estimation (MPLE) method and the Markov chain Monte Carlo maximum likelihood estimation (MCMC MLE) method. The MPLE method transfers the model into logit form, and then applies logistic regression techniques to conduct likelihood fit tests. The core of MCMC MLE is designed to simulate random graph distribution from a set of parameter values. It adjusts parameter values by comparing the distributions of corresponding random graphs and observed graphs, and then repeats the process until the estimated value becomes stable. Studies have shown that the MCMC MLE method works better than the MLE method, especially when the network has a strong dyadic dependence.

### Statistical Analyses

Statnet’s ERGM R software package was used to conduct statistical analysis. The study applied MCMC MLE to conduct parameter estimations. First, peer relationship networks in each class were built according to the measurement of peer networks (Lubbers and Snijders, 2007). They were then stored in relational data files by matrix form. To build a relationship network, each student in the class was regarded as a network actor; the connections between them formed the relationships in the network. The relationship network in each class was composed of a square matrix, in which the rows and columns were all students in the network. The elements/data in the square matrix represented whether there were connections between students. If a student nominated another student, this indicated they had a connection which was recorded as 1; otherwise, it would be recorded as 0. The square matrix was not symmetrical; that is, although A nominated B, B might not nominate A. The attribute data files for each network were then built according to demography variables. The data files were in SPSS format. A list of data corresponded to an attribute variable. Having built an attribute data file, all individual attribute variables needed to be standardized in order to compare the data. For the initial ERGM model, the effects of each attribute variable were assessed separately. Finally, the three dimensions of subjective well-being were brought into the model to build the final ERGM, and the effects of multiple attribute variables assessed simultaneously.

In order to control structure effects when inspecting attribute variables effects, the model contained both attribute variables effects and structure effects. To further explore the mode of peer relationships in the network, the study incorporated four common structure effects: namely, reciprocal structure, transitivity structure, popularity structure and expansiveness structure. With regard to attribute variables effects, in order to test Hypothesis 5 the study considered differential effects. Differential effects were based on the absolute difference in some attribute variables between individuals who had relations with each other. If the estimated result of the differential effects parameter was negative and had statistical significance, it showed that, under the invariable condition of other effects in the model, individuals with relations tended to have similarities in their attribute variables. In order to test Hypotheses 6 and 7, the study also estimated receiver effect parameters, which were based on the interactions between attribute variables and network structures. The study also analyzed the relationship between subjective well-being and popularity structure. If there was a positive correlation between them, it indicated that an individual with high scores in SWB would be more popular.

## Statistical Results

### Model Fitting Degree

The *t-ratio* is defined as the estimate of a parameter divided by its standard error, with reference to a standard normal null distribution (Snijders, 2001), and it is often applied to balance the fitting degree of each parameter, which is calculated by taking the observed values minus the sample mean, then dividing by the standard error. **Table 2** shows the estimated *t-ratio* of each parameter in our ERGMs, which were based on the relationship networks in each class. Every parameter of the ERGM in the 15 networks was between -1 and 1, which indicates that the model built from the 15 classes was an acceptable fit that reflected the features of the network.

**Table 2:** The estimated *t-ratio* of each parameter in the exponential random graph model.

Class	Reciprocity	Popularity	Expansiveness	Transitivity	Life Satisfaction	Positive Affection	Negative Affection
Class A	0.47	0.04	-0.44	-0.11	0.08	-0.02	0.20
Class B	0.95	0.05	0.05	0.60	-0.07	-0.24	0.08
Class C	0.58	-0.18	-0.25	0.04	-0.13	-0.05	-0.24
Class D	0.18	-0.33	-0.55	-0.55	-0.21	0.02	-0.08
Class E	0.26	-0.38	-0.28	-0.30	0.11	0.02	0.10
Class F	0.54	-0.16	-0.33	-0.01	-0.38	-0.09	0.03
Class G	0.70	-0.01	-0.04	0.15	-0.33	-0.17	0.16
Class H	0.92	-0.23	-0.41	0.17	-0.20	-0.06	-0.10
Class I	0.76	-0.33	-0.65	-0.23	-0.10	-0.25	-0.19
Class J	0.98	0.00	-0.08	0.20	-0.02	0.15	0.17
Class K	0.36	-0.14	-0.49	-0.31	-0.42	-0.04	0.03
Class L	0.89	-0.16	-0.16	0.09	-0.22	-0.18	-0.17
Class M	0.45	-0.33	-0.42	-0.20	-0.03	-0.08	-0.18
Class N	1.00	-0.04	-0.25	0.33	-0.13	-0.15	-0.02
Class O	0.79	0.02	-0.13	0.09	-0.13	-0.06	-0.01


### Structure Effects

The estimated values and standard errors of network structure effects are presented in **Table 3**. The results show the following:

**Table 3:** The estimated values (standard errors) of network structure effects.

Class	Reciprocity	Transitivity	Popularity	Expansiveness
Class A	4.14 (0.27)^∗∗^	2.04 (0.15)^∗∗^	0.02 (0.03)	-2.14 (0.19)^∗∗^
Class B	4.07 (0.20)^∗∗^	1.46 (0.10)^∗∗^	-0.03 (0.02)	-0.67 (0.06)^∗∗^
Class C	3.31 (0.24)^∗∗^	1.02 (0.16)^∗∗^	-0.04 (0.02)	-0.43 (0.06)^∗∗^
Class D	3.73 (0.33)^∗∗^	1.41 (0.14)^∗∗^	-0.11 (0.06)*	-0.85 (0.13)^∗∗^
Class E	3.05 (0.30)^∗∗^	1.14 (0.13)^∗∗^	-0.03 (0.03)	-0.47 (0.08)^∗∗^
Class F	3.42 (0.30)^∗∗^	1.29 (0.14)^∗∗^	0.02 (0.01)	-0.76 (0.09)^∗∗^
Class G	3.31 (0.28)^∗∗^	1.29 (0.11)^∗∗^	-0.08 (0.04)*	-0.47 (0.07)^∗∗^
Class H	3.12 (0.36)^∗∗^	0.78 (0.38)^∗^	-0.24 (0.13)	-1.01 (0.26)^∗∗^
Class I	3.75 (0.33)^∗∗^	1.29 (0.22)^∗∗^	-0.01 (0.04)	-1.18 (0.18)^∗∗^
Class J	3.50 (0.31)^∗∗^	1.37 (0.21)^∗∗^	0.02 (0.02)	-1.31 (0.15)^∗∗^
Class K	2.74 (0.25)^∗∗^	1.09 (0.07)^∗∗^	-0.01 (0.01)	-0.30 (0.03)^∗∗^
Class L	3.13 (0.33)^∗∗^	1.04 (0.17)^∗∗^	-0.24 (0.08)**	-0.18 (0.08)^∗^
Class M	2.84 (0.26)^∗∗^	0.85 (0.13)^∗∗^	-0.11 (0.03)**	-0.12 (0.03)^∗∗^
Class N	3.56 (0.28)^∗∗^	1.90 (0.18)^∗∗^	-0.03 (0.05)	-1.92 (0.21)^∗∗^
Class O	3.45 (0.33)^∗∗^	1.42 (0.16)^∗∗^	-0.00 (0.03)	-0.86 (0.13)^∗∗^


*^∗^*p* < 0.05, ^∗∗^*p* < 0.01.*

(1) *Reciprocal effect*: The 15 networks all had obvious reciprocal effects, which meant individuals tended to select each other as friends.
(2) *Transitivity effect*: The transitivity parameters of the 15 relationship networks were all greater than 0.05 and had statistical significance, which meant a friend’s friend tended to be a friend.
(3) *Popularity effect*: The popularity effects in most classes were not obvious, which meant the in-degree (the total number of actors selecting an individual) of individuals in the class networks had little difference. The distribution of relationships was average. Among them, the parameters of popularity effects in classes D, G, L, and M were negative and had statistical significance, which showed that the actual appearance probability of popularity structure was lower than that of a random level in the four networks.
(4) *Expansiveness effect*: All classes presented obvious negative expansiveness effects, which showed that an individual’s social circle was stationary in the network; i.e., an individual would not take the initiative to make friends with people outside the circle.

### Differential Effects

Absolute differential effect parameters were applied to inspect the assumption “Adolescent peers have similarities in subjective well-being.” If the estimated results were negative and had statistical significance, then they supported the assumption. The estimated results of differential effect parameters for the initial model (the effects of each attribute variable were separately assessed) and the final model (the effects of multiple attribute variables were simultaneously assessed) are shown in **Table 4**. The final model shows adolescent friends had some obvious similarity tendencies in each dimension of subjective well-being.

**Table 4:** The estimated values of differential effects parameters.

Class	Life satisfaction		Positive affection		Negative affection	
			
	Initial	Final	Initial	Final	Initial	Final
Class A	-**0.16**	-0.12	**0.75**	-**0.15**	-0.10	-0.08
Class B	-**0.18**	-0.10	-**0.14**	-**0.13**	-**0.26**	-**0.25**
Class C	-0.09	-0.04	-0.13	-0.12	-0.06	-0.05
Class D	0.14	0.15	-0.08	-0.07	-0.02	0.10
Class E	-**0.32**	-**0.30**	-**0.18**	-**0.15**	-**0.20**	-**0.18**
Class F	-0.09	-0.10	-0.14	-0.13	-0.13	-0.12
Class G	-**0.22**	-**0.20**	-**0.18**	-0.14	-0.10	-0.08
Class H	-**0.34**	-**0.30**	-0.10	-0.03	-**0.30**	-**0.24**
Class I	-0.16	-0.14	-0.07	-0.07	0.01	0.03
Class J	0.02	0.02	-0.01	0.01	0.03	0.03
Class K	-**0.21**	-**0.15**	-**0.16**	-0.11	-**0.22**	-**0.21**
Class L	-**0.34**	-**0.26**	-**0.25**	-**0.20**	-**0.24**	-**0.24**
Class M	-**0.25**	-0.15	-**0.44**	-**0.35**	-**0.25**	-**0.17**
Class N	-0.12	-0.12	-0.10	-0.06	-**0.19**	-**0.17**
Class O	-0.12	-0.07	-**0.18**	-0.16	-0.18	-0.12


*The black font indicates that the results of parameter estimations are obvious.*

In the initial model which only considered satisfaction with life, the differential effect parameters of eight networks A, B, E, G, H, K, L, and M were negative and had statistical significances. Among them, when considering the three dimensions of subjective well-being in the final model, the differential effects of five classes E, G, H, K, and L were negative and had statistical significances, which shows that there was a similar satisfaction with life among friends in five networks. A, B, and M did not have statistical significances in the final model, although the initial model showed a similar satisfaction with life among friends in the three networks. The observation that they did not have similarities when affected by other variables in the final model shows that individuals in the networks might not form friendships based on the similarity of satisfaction with life but on other correlated variables. The differential effects in the other seven networks were not obvious.

For positive affective dimensions, only the differential effect parameters of eight networks A, B, E, G, K, L, M, and O in the initial model were negative and had statistical significances. Among them, when simultaneously considering three dimensions of subjective well-being in the final model, the differential effects of five classes A, B, E, L, and M were also negative and had statistical significances, which showed that there were similar positive affects among friends in five networks. G, K, and O did not have statistical significances in the final model although, in the initial model, there were similar positive affective levels among friends in these three networks. The observation that they did not have similarities when affected by other variables in the final model shows that individuals in these networks might not form friendships based on the similarity of positive affects but on other correlated variables. The differential effects in the other seven networks were not significant.

For negative affective dimensions, the differential effect parameters of seven networks B, E, H, K, L, M, and N in the initial model were negative and had statistical significances. Among them, when simultaneously considering three dimensions of subjective well-being in the final model, the differential effects of all seven classes B, E, H, K, L, M, and N were also negative and had statistical significances, which shows that there were similar negative affects among friends in these seven networks. The differential effects in the other eight networks were not significant.

### Receiver Effects

Receiver effects are based on the relationship between subjective well-being and the popularity of individual attribute variables. The results of receiver effects in each relationship network are shown in **Table 5**. Only life satisfaction and popularity in class D and class M had significantly positive correlations (*r* = 0.39, *p* < 0.05; *r* = 0.41, *p* < 0.05), which showed that an individual with higher satisfaction with life would be more popular.

**Table 5:** The estimated results of receiver effect parameters.

Class	Life satisfaction	Positive affection	Negative affection
Class A	0.08	0.07	0.06
Class B	0.18	0.20	0.11
Class C	0.26	0.22	0.29
Class D	0.39^∗^	0.25	0.27
Class E	0.15	0.15	0.19
Class F	0.14	0.07	0.08
Class G	0.25	0.23	0.28
Class H	0.28	0.26	0.24
Class I	0.19	0.20	0.22
Class J	0.17	0.13	0.14
Class K	0.01	0.19	0.04
Class L	0.28	0.27	0.27
Class M	0.41^∗^	0.21	0.28
Class N	0.13	0.11	0.09
Class O	0.21	0.16	0.16


*^∗^*p* < 0.05.*

## Conclusion and Discussion

This study examined the network features of adolescent peer relationships and then applied ERGMs to inspect the impacts of adolescent peer relationships on subjective well-being. As regards reciprocal effects, transitivity effects and expansiveness effects, the findings were in line with previous research on peer relationship networks. However, the findings on receiver effects and differential effects show interesting differences from previous studies. We discuss each of these in turn.

### Reciprocal Effects

All the peer relationship networks in our study showed positive reciprocal effects. Reciprocity is the most fundamental and common behavior in human activities (Blau, 1964). It is also a significant part of friendship and plays a significant role in friends’ selections (Snyder et al., 1996). Adolescents are no exception. Mutual friends have more opportunities to influence each other and form similarities between each other (Mercken et al., 2010). A reciprocal relationship also improves the quality of friendship and enhances intimacy (Hinde et al., 1985; Dishion et al., 1996). Reciprocity is a major feature of adolescent friendship (Rubin et al., 1998), and a reciprocal relationship between friends is the significant factor in adolescent peer groups (Pearson and Michell, 2000).

### Transitivity Effects

All the peer relationship networks in our study showed positive transitivity effects: that is, my friend’s friend might become my friend. The results confirm previous findings on adolescent friend networks (Espelage et al., 2007). The main reason why transitivity exists in a network is that the actors attempt to reduce the contradictions and uncertainties in social and cognitive situations and make efforts to establish a balance in interpersonal relationships. In a tripartite relation between friends, for instance, unbalanced relations occur when actor E likes actor R, actor R likes actor V, but actor E does not like actor V. This might cause emotional stress and uncertainty (Batjargal, 2007). Therefore, adolescents might tend to build transitive relations with other peers to establish an equilibrium in a tripartite relationship.

### Expansiveness Effects

All the peer relationship networks in our study showed negative expansiveness effects. The network circles in a class are relatively stationary. The reason is because adolescence is a psychologically sensitive period and adolescents fear rejection, so they have a lower initiative to make friends. Meanwhile, in order to maintain a stable friendship and optimize groups, the individual relationship circles are often exclusive, which makes it difficult for people outside the circle to enter and leads to less volatility for each circle.

### Leaders and Receiver Effects

The analysis of popularity effects indicates that the peer relationship networks in our study have no leaders and have no significant receiver effects. No leader emergence might be due to the fact that the adolescents surveyed in our investigation all came from urban schools and do not live in the school (as is the case with boarding schools). Learning is the main task for adolescents and they have little distractions beyond learning, which means there is little ground for the emergence and growth of adolescent leaders. Besides, the generations after 2000 have grown into adolescence in the Internet age. Social media networks expand their social horizons and enhance their cognitive levels which, to a certain extent, would obviously hinder the emergence of leaders in adolescent peer networks.

Meanwhile, whilst our study found that receiver effects were not significant, there were no correlations between popularity in the network and each dimension of subjective well-being among most friends. There might be two reasons for this. Firstly, popularity was the result of evaluation from others, while subjective well-being was self-evaluated. Secondly, subjective well-being has multiple sources, while the classroom environment is only one factor.

### Differential Effects

The results of differential effects show that adolescents in partial peer networks in our study exhibit similar levels in SWB dimensions (satisfaction with life, positive affects and negative affects), which is consistent with the results of previous studies (Ryan, 2001), but not all network friends presented such similarities. The possible reasons for these mixed results are as follows.

(1) Our study overcame the lack of peer self-reports in previous studies which increases the objectivity and credibility of the results. In previous studies, participants were only required to report the attribute variables of their friends. Adolescents might overestimate the similarities among friends. In our study, all individuals in the network were required to do self-reporting so that the results would be more reliable.
(2) The structural features in this study were different from previous studies, and therefore different results are to be expected. Previous studies have been based on binary relationship structures (such as reciprocal structures) while this study was based on structural features such as transitivity, expansiveness, and popularity. These ternary or multiple structures were constituted by the interdependence of multiple dual structures which are closer to real situations and have a greater practical significance. The results therefore are more reliable and have a greater accuracy.
(3) This investigation studied the variables of peer relationships only and did not take into account other factors which could affect the conclusions (see below).

### Subjective Well-being and Future Research

Studies have shown that subjective well-being among friends will lead to an effect on each other and tends to reach a similar level (Fowler and Christakis, 2008). Social Communication Theory and Similarity Theory could explain the similarities among friends. For instance, these similarity effects might be the result of social influences: that is, adolescents are affected by their friends and take on similar behaviors. But they might also be the result of social choice: that is, forming friendships based on similar attitudes and behaviors such as a similar SWB. Whether the similarities among adolescents are the result of social communication and influences or social choices needs further exploration, and future research could investigate the potential processes of SWB similarities among adolescents by longitudinal studies.

This study shows that peer networks can affect an individual’s subjective well-being. The peer environment in school plays a role in the process of maintaining groups of friends. However, individual behaviors in the groups tend to promote each individual and tend to be consistent. Since similar friends gather together into smaller peer groups, so we could apply group counseling to intervene in specific groups of friends. We could also through influential individuals to intervene with other peer in the network, by which we could promote the healthy development of the individual and ultimately promote social progress.

From the methodological point of view, this study adds to the current literature on ERGMs, and also provides a platform for future research. As one of our social network analysis models, ERGMs are concerned not only with the relationship between individual and individual, but also with a more in-depth study of the dependent relationships between individuals. One advantage of an ERGM is the ability to apply a simple graphical structure to present selected local structure variables. Additionally, the selection of structure variables is quite flexible and can easily be corrected. A further advantage is that the potential statistics in an ERGM enables a more in-depth exploration of the dependent relationships between individuals compared to other network models. (Hossain et al., 2015). In future, it could be extended from binary random variables to classified relational variables or multiple relational variables. It could also be put into use in the fields of sociology, economics, and psychology and promote interdisciplinary collaboration in the study of peer relationships.

## Limitations

There are some aspects of our study that need to be improved: Firstly, we did not measure other variables that could influence the results such as age, gender and social economic level. Secondly, we cannot completely exclude the possibility that the lack of a relationship between popularity and subjective well-being (the receiver effect) is related to the lack of a popularity structure effect. Thirdly, longitudinal research is needed to explore the potential changes in the similarity effect of peers’ subjective well-being. The similarity effect may be the result of social influence, as adolescents can be influenced by their friends and act out similar behaviors and performance; but it may also be the result of social choice, as adolescents can select friends on the basis of similar behaviors and attitudes such as subjective well-being.

## Author Contributions

CJ, FC, JL, and XP developed the concepts for the study. HW collected the data. CJ, TW, JL, XP, and HW analyzed the data. CJ, JL, XP, and HW wrote the manuscript. All authors contributed to the manuscript and approved the final version of the manuscript for submission.

## Conflict of Interest Statement

The authors declare that the research was conducted in the absence of any commercial or financial relationships that could be construed as a potential conflict of interest. The reviewer MI-C and handling Editor declared their shared affiliation, and the handling Editor states that the process nevertheless met the standards of a fair and objective review.
